# Accumulation of the solvent vehicle sulphobutylether beta cyclodextrin sodium in critically ill patients treated with intravenous voriconazole under renal replacement therapy

**DOI:** 10.1186/1472-6904-6-6

**Published:** 2006-09-18

**Authors:** Marc A von Mach, Jürgen Burhenne, Ludwig S Weilemann

**Affiliations:** 1 Division of Intensive Care Medicine and Clinical Toxicology, II. Medical Department, University of Mainz, Langenbeckstr. 1, 55131 Mainz, Germany; 2 Department of Internal Medicine VI, Clinical Pharmacology and Pharmacoepidemiology, University of Heidelberg, Im Neuenheimer Feld 410, 69120 Heidelberg, Germany

## Abstract

**Background:**

Voriconazole was introduced for the treatment of life-threatening fungal infections. The intravenous form includes the solvent vehicle sulphobutylether beta cyclodextrin sodium which shows an impaired clearance under intermittent dialysis therapy. This investigation aimed to determine first clinical data on sulphobutylether beta cyclodextrin sodium blood levels to verify the risk for accumulation.

**Methods:**

In four patients suffering from renal insufficiency and intermittent dialysis therapy who needed a treatment with intravenous voriconazole as a reserve antifungal at the intensive care unit of the Mainz University Hospital the trough levels of voriconazole and sulphobutylether beta cyclodextrin sodium were measured.

**Results:**

A 75-year-old woman showed a maximal sulphobutylether beta cyclodextrin sodium plasma level of 145 μg/ml in the initial phase. After a few days renal function recovered and the plasma levels came down to less than 20 μg/ml. In contrast to this patient with a recovery of renal function the remaining three patients showed renal failure during the complete period of intravenous treatment with voriconazole. In these patients an accumulation of sulphobutylether beta cyclodextrin sodium plasma levels was determined with a maximum of 523 μg/ml in a 18-year-old man, 409 μg/ml in a 57-year-old man, and 581 μg/ml in a 47-year-old man.

**Conclusion:**

The present data indicate an accumulation of sulphobutylether beta cyclodextrin sodium in patients treated with intravenous voriconazole and dialysis therapy. Fortunately, no toxic effects were observed, although the accumulated dose values were lower but comparable with those used in previous toxicity studies with animals.

## Background

The triazole antifungal, voriconazole, was introduced for the treatment of life-threatening fungal infections. The drug, which is available for both oral and intravenous administration, has broad-spectrum activity against pathogenic yeasts, dimorphic fungi and opportunistic moulds [1,2]. As voriconazole has limited aqueous solubility, the intravenous form includes the solvent vehicle sulphobutylether beta cyclodextrin sodium as a novel delivery system [3]. In healthy subjects sulphobutylether beta cyclodextrin sodium is rapidly eliminated with a terminal half-life of 1.6 hours. The clearance of sulphobutylether beta cyclodextrin sodium is linearly related to creatinine clearance and accumulation has been described in subjects with moderate to severe renal impairment (serum creatinine levels > 2.5 mg/dl) [4]. In patients with an estimated creatinine clearance of 30 to 50 ml/min the mean Cmax and AUC of sulphobutylether beta cyclodextrin sodium increased by almost 50% and 4-fold, respectively, compared to subjects with normal renal function [5]. Additionally, sulphobutylether beta cyclodextrin sodium is known to show an impaired clearance under dialysis therapy of 55 ml/min determined in volunteers with renal failure undergoing hemodialysis [6]. Clinical data regarding this problem have not been available so far. The manufacturer suggests to balance the possible risk of accumulation of sulphobutylether beta cyclodextrin sodium and the benefit of the antifungal effect [7]. Furthermore, it is recommended to treat patients on dialysis therapy only with the oral form of voriconazole if feasible. However, in critically ill patients with complicated life-threatening fungal infections a save oral administration of drugs is difficult to accomplish as, for instance, extensive gastric reflux, gastrointestinal bleedings or mucositis are frequent comorbidities. In animal experiments the acute toxicity of sulphobutylether beta cyclodextrin sodium has been observed to be low. The minimal lethal dose was 2000 mg/kg. Target organs for toxic effects in rodents were kidney and liver with obstruction of renal tubules and single cell necrosis in the liver. Both findings were a consequence of massive cytoplasmic vacuolation [4]. In two healthy volunteers peak sulphobutylether beta cyclodextrin sodium levels of 118 and 130 μg/ml, respectively, were measured immediately following administration of 1600 mg sulphobutylether beta cyclodextrin sodium as a single 15 minutes intravenous infusion [8]. The present investigation aimed to determine first clinical data on sulphobutylether beta cyclodextrin sodium blood levels to verify the risk for accumulation.

## Methods

### Subjects

From March 2003 to January 2006 a total number of 29 critically ill patients (17 male, 12 female) needed a treatment with intravenous voriconazole as a reserve antifungal at the 10-bed general intensive care unit of the Mainz University Hospital. 15 patients (51.7 %) required intermittent dialysis due to acute (14 patients) or chronic (1 patient) renal failure. In four patients suffering from renal insufficiency the trough plasma levels of voriconazole and sulphobutylether beta cyclodextrin sodium were measured. Blood samples were collected in Monovette plastic tubes and immediately centrifuged at 2000*g *at 4°C. This investigation was approved by the Institutional Review Board (Rheinland-Pfalz, Germany). The board waived the need for informed consent. Additionally, monitoring for possible toxic effects of sulphobutylether beta cyclodextrin sodium regarding level of consciousness, hemodynamic stability, dermal reactions, and liver function tests was performed.

### Voriconazole and sulphobutylether beta cyclodextrin sodium measurements

Voriconazole was determined according to a previously published method [9]. In brief, plasma samples were spiked with internal standard and borate buffer was added. The samples were loaded onto solid phase extraction (SPE) columns, washed with buffer and methanol/water, and eluted with methanol/acetic acid mixture. After drying, the extracts were reconstituted with mobile HPLC phase and analyzed by HPLC/UV. The limit of quantification was 0.2 μg/ml. The precision of determination (coefficient of variation) was 7.2, 7.9, 3.2, and 3.7% at quality control concentrations of 0.2, 0.4, 3.4, and 6.9 μg/ml, respectively. Sulphobutylether beta cyclodextrin sodium was determined according to a previously published method [8]: Phosphate buffer was added to plasma samples before loading onto SPE columns (Chromabond, cyclohexyl). The loaded SPE columns were washed with buffer and eluted with methanol/water (30/70) mixture. After drying, the extracts were reconstituted in methanol/water (10/90) and analyzed by HPLC/Fluorescence. The limit of quantification was 4.00 μg/ml. The precision of determination (coefficient of variation) was 5.7, 6.7, 7.0, and 6.9% at quality control concentrations of 4.00, 14.9, 65.2, and 126 μg/ml, respectively. The analytical methods were validated according to FDA validation guidelines and fulfilled the respective quality assurance for accuracy and precision.

## Results

The analysis of the four patients suffering from renal insufficiency for whom plasma levels of voriconazole and sulphobutylether beta cyclodextrin sodium were measured revealed two different courses of renal impairment which required a clear differentiation:

### Patients with restoration of renal function

**Figure 1:**
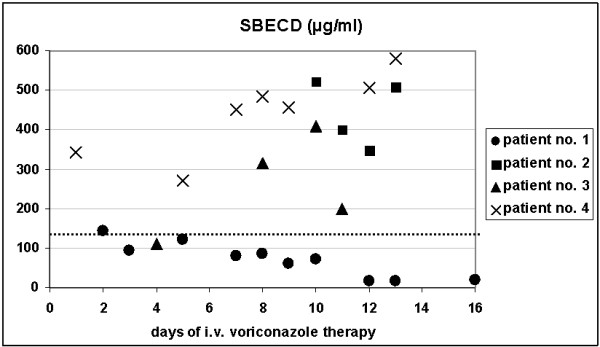
**Sulphobutylether beta cyclodextrin sodium (SBECD) levels in patients on intermittent dialysis receiving intravenous voriconazole**. Patients no. 2–4 with acute renal failure without recovery of renal function under intermittent dialysis receiving intravenous voriconazole show higher levels of sulphobutylether beta cyclodextrin sodium (SBECD) as compared to patient no. 1 with recovery of renal function. For comparison, in healthy volunteers peak SBECD levels of 130 μg/ml (dotted line) were measured immediately following administration of 1600 mg SBECD as a single infusion [9].

A 75-year-old woman submitted with acute myeloid leukemia showed a maximal sulphobutylether beta cyclodextrin sodium plasma level of 145 μg/ml in the initial phase of treatment with intravenous voriconazole (Figure 1, Table 1, patient no. 1). After a few days renal function recovered and the plasma levels came down to less than 20 μg/ml after 10 days of treatment with intravenous voriconazole.

**Table 1:** Voriconazole (VOR) and sulphobutylether beta cyclodextrin sodium (SBECD) plasma levels in patients with intravenous voriconazole therapy and renal failure

patient no.	day of VOR therapy	dose of intravenous VOR	dose of SBECD	dialysis information	VOR (μg/ml)	SBECD (μg/ml)
1	2	2 × 400 mg	2 × 6400 mg	before dialysis	0.9	98.3
1	2	2 × 200 mg	2 × 3200 mg	after dialysis	1.1	145
1	3	2 × 200 mg	2 × 3200 mg	before dialysis	0.2	93.3
1	4	2 × 200 mg	2 × 3200 mg	after dialysis	< 0.2	121
1	7	2 × 200 mg	2 × 3200 mg	after dialysis	0.4	78.9
1	8	2 × 200 mg	2 × 3200 mg	before dialysis	0.5	86.4
1	9	2 × 200 mg	2 × 3200 mg	before dialysis	0.4	59.6
1	9	2 × 200 mg	2 × 3200 mg	after dialysis	< 0.2	31.4
1	10	2 × 200 mg	2 × 3200 mg	before dialysis	0.3	71.6
1	12	2 × 200 mg	2 × 3200 mg	no dialysis	< 0.2	15.6
1	13	2 × 200 mg	2 × 3200 mg	no dialysis	< 0.2	16.4
1	16	2 × 200 mg	2 × 3200 mg	no dialysis	< 0.2	18.4
2	10	2 × 200 mg	2 × 3200 mg	before dialysis	0.4	523
2	11	2 × 200 mg	2 × 3200 mg	after dialysis	0.3	400
2	12	2 × 200 mg	2 × 3200 mg	no dialysis	0.3	348
2	13	2 × 200 mg	2 × 3200 mg	no dialysis	0.7	508
3	4	2 × 400 mg	2 × 6400 mg	no dialysis	3.8	110
3	8	2 × 400 mg	2 × 6400 mg	before dialysis	5.1	314
3	10	2 × 400 mg	2 × 6400 mg	after dialysis	3.2	409
3	11	2 × 400 mg	2 × 6400 mg	after dialysis	2.6	200
4	1	2 × 400 mg	2 × 6400 mg	before dialysis	1.4	344
4	5	2 × 200 mg	2 × 3200 mg	before dialysis	0.3	271
4	7	2 × 200 mg	2 × 3200 mg	before dialysis	0.3	451
4	8	2 × 200 mg	2 × 3200 mg	after dialysis	0.3	483
4	9	2 × 200 mg	2 × 3200 mg	no dialysis	0.9	456
4	12	2 × 200 mg	2 × 3200 mg	after dialysis	1.1	505
4	13	2 × 200 mg	2 × 3200 mg	before dialysis	1.3	581
4	13	2 × 200 mg	2 × 3200 mg	after dialysis	0.9	563

### Patients without restoration of renal function

In contrast to the patient with a recovery of renal function the remaining three patients showed renal failure during the complete period of intravenous treatment with voriconazole. In these patients an accumulation of sulphobutylether beta cyclodextrin sodium plasma levels was determined with a maximum of 523 μg/ml in a 18-year-old man submitted with myocarditis (Table 1, patient no. 2), 409 μg/ml in a 57-year-old man submitted with multiple myeloma (Table 1, patient no. 3), and 581 μg/ml in a 47-year-old man submitted with intestinal ischemia (Table 1, patient no. 4).

### Voriconazole plasma levels

Besides sulphobutylether beta cyclodextrin sodium voriconazole plasma levels were determined which were most commonly less than 1.5 μg/ml and showed no evidence for accumulation (Table 1). In patient no. 1 the levels were, in part, not detectable. In patient no. 3 all voriconazole measurements were distinctly elevated as compared to the remaining patients which was due to a concomittant hepatic insufficiency.

### Technical aspects of dialysis, clinical and biochemical monitoring

Hemodialysis in the four patients was performed using a low flux AM-BIO 750 cartridge (ASAHI KASEI MEDICAL EUROPE GmbH, Frankfurt, Germany). The average frequency of the dialysis sessions was every 24 to 48 hours, the duration was 4 hours, blood flow 200 to 220 ml/min, and fluid extraction 2 to 3 liters to maintain proper fluid balance. Clinical and biochemical data to assess the toxicity of accumulation of sulphobutylether beta cyclodextrin sodium were determined but in these critically ill patients with complicated disease it was difficult to trace back possible effects. Patient no. 3 was sedated, intubated and mechanically ventilated during the whole course of intravenous voriconazole treatment. Patients no. 2 and 4 were awake but tracheotomized and not fully orientated. Patient no. 1 was extubated but she was somnolent and could not answer questions. None of the patients showed an obvious deterioration of the level of consciousness during the treatment with voriconazole. A more detailed assessment like testing for the frequently described side effect of visual disturbances under intravenous voriconazole could not be performed in these patients. Dermal reactions were not observed under intravenous voriconazole treatment. Patients no. 1, 2, and 4 were hemodynamically stable without catecholamines. Patient no. 3 needed treatment with noradrenaline which required adjustment to individual situations like during high fever or during dialysis. Liver function test results at the beginning of intravenous voriconazole treatment compared to the end of treatment showed the hepatic impairment of patient no. 3 (Table 2).

**Table 2:** Liver function tests in patients with renal failure at start and end of intravenous voriconazole therapy

	ASAT (IU/l)^*a*^		γ-GT (IU/l)^*b*^		Total bilirubin (mg/dl)^*c*^	
	start	end	start	end	start	end

patient no. 1	104	24	119	264	2.32	1.39
patient no. 2	100	26	93	71	0.83	0.32
patient no. 3	54	25	288	443	4.93	7.27
patient no. 4	30	22	238	216	3.29	1.34

^*a*^norm < 37 IU/l, ^*b*^norm 10–66 IU/L, ^*c*^norm 0.1–1.2 mg/dl

## Discussion

Patients with the need for an intravenous treatment with voriconazole were rare with about 10 patients per year at the 10-bed general intensive care unit. However, when such a treatment was required more than half of the patients were under renal replacement therapy.

### Sulphobutylether beta cyclodextrin sodium findings

The present data indicate an accumulation of sulphobutylether beta cyclodextrin sodium in patients with renal failure and intermittent dialysis therapy given intravenous voriconazole in whom restoration of renal function was not achieved during the treatment course. In contrast, in the patient in whom restoration of renal function was obtained early during the treatment with intravenous voriconazole, accumulation of sulphobutylether beta cyclodextrin sodium was not observed. Fortunately, there was no evidence for toxic effects related to the concentrations of sulphobutylether beta cyclodextrin sodium measured in these patients. Assuming no abnormalities in the volume distribution (the steady-state volume of distribution of sulphobutylether beta cyclodextrin sodium is approximately 0.2 L/kg, which is similar to extracellular fluid volume in humans) the maximal concentration values of sulphobutylether beta cyclodextrin sodium would be translate back to a total amount of about 100 mg/kg accumulated sulphobutylether beta cyclodextrin sodium on board. This extrapolated dose value was about 10-fold lower than the usual doses used in animal models for toxicity assessment. In animal experiments the minimal single lethal dose has been determined to be 2000 mg/kg, while daily doses of 1000 mg/kg for one month or 600 mg/kg for six months did not produce functional renal changes [4]. Assuming a half-life of sulphobutylether beta cyclodextrin sodium in healthy animals of 1.6 hours the single doses of 1000 or 600 mg/kg would have been eliminated within a few hours (animals receiving 1000 mg/kg would have less than 100 mg/kg on board within about 3.5 hours). In contrast, the 100 mg/kg in our patients with renal failure would represent a continuous baseline level. Therefore, the range of the presently determined sulphobutylether beta cyclodextrin sodium concentrations might well be considered to be toxic from clinical perspective. Nevertheless, for comparisons with animal data a word of caution is suitable regarding the presently investigated patients with renal insufficiency. The sick kidney may be protected from the toxic action of sulphobutylether beta cyclodextrin sodium, which is nephrotoxic through its reuptake from glomerular filtrate by the kidney tubules. This phenomenon has been shown for aminoglykoside antibiotics, which share this mechanism of toxicity. However, accumulation of drug delivery vehicles bare many potential risks to these critically ill patients and should be prevented [10,11]. Alternative antifungals like liposomal amphotericin B or caspofungin should be considered or intravenous voriconazole therapy should be switched to oral administration as soon as possible. The possible accumulation of sulphobutylether beta cyclodextrin sodium in patients under renal replacement therapy should be studied in prospective clinical studies in more detail. Furthermore, different renal replacement techniques like dialysis with high-flux membranes or continuous veno-venous hemofiltration need to be evaluated regarding the clearance of sulphobutylether beta cyclodextrin sodium.

### Voriconazole findings

Regarding the voriconazole plasma levels published data in patients with renal insufficiency is rare. A case report in a patient with chronic renal insufficiency treated by continuous veno-venous hemodiafiltration, which focussed solely on voriconazole levels, demonstrated that no dosage adjustment was required [12]. The voriconazole plasma levels presently found in patients no. 1, 2, and 4 were in line with published data on healthy subjects which received 3 mg kg-1 twice daily. By day 12 this previous study showed a maximal concentration of voriconazole of 2.4 μg/ml and a half-life of 6.4 hours [13]. The trough levels of voriconazole presently determined were at several points lower than the minimum inhibitory concentrations for aspergillus spp., candida spp., and for most emerging fungal pathogens (minimal concentration > 0.8 μg/ml). Nevertheless, voriconazole treatment levels < 0.2 μg/ml have frequently been observed in successfully treated patients and the manufacturer does not suggest a dosage adjustment [7]. The higher levels of voriconazole in patient no. 3 were in line with our previous observations that patients with liver insufficiency frequently show levels around 5 μg/ml (our valid range for voriconazole plasma levels is from 0.2 to 10 μg/ml). Previous studies showed that liver function test abnormalities were statistically significant but weakly associated with elevated plasma voriconazole concentrations. To investigate potential threshold effects, patients with plasma voriconazole concentrations above and below various threshold values (1, 2, 3, 4, 5, 6 μg/ml) were analyzed. These investigations did not identify threshold plasma concentrations above which the risk of a liver function test abnormality was higher compared with plasma concentrations below the threshold [4].

## Conclusion

The present data indicated an accumulation of sulphobutylether beta cyclodextrin sodium in patients treated with intravenous voriconazole and dialysis therapy. Obvious adverse effects related to the accumulation of the drug delivery vehicle could not be observed, although the presently extrapolated dose values were lower but comparable with those used in animal experiments for toxicity studies. Intravenous application of voriconazole in patients on dialysis therapy should be evaluated carefully. Systematic clinical investigations are required to prevent potential harm to these critically ill patients due to an accumulation of sulphobutylether beta cyclodextrin sodium.

## Competing interests

MAvM received speaking fees of 500 Euro from the manufacturer of voriconazole (Pfizer Pharma GmbH, Karlsruhe, Germany). JB and LSW have no financial or non-financial competing interest related to the content of the manuscript.

## Authors' contributions

MAvM treated the patients at the intensive care unit, conceived the investigation, collected the blood samples and drafted the manuscript. JB carried out the voriconazole and sulphobutylether beta cyclodextrin sodium analyses and helped to draft the manuscript. LSW treated the patients at the intensive care unit and participated in the coordination of the investigation. All authors read and approved the final manuscript.

## Pre-publication history

The pre-publication history for this paper can be accessed here:


